# The role of NLRP3 inflammasome in 5-fluorouracil resistance of oral squamous cell carcinoma

**DOI:** 10.1186/s13046-017-0553-x

**Published:** 2017-06-21

**Authors:** Xiaodong Feng, Qingqiong Luo, Han Zhang, Han Wang, Wantao Chen, Guangxun Meng, Fuxiang Chen

**Affiliations:** 1 Department of Clinical Immunology, Ninth People’s Hospital, Shanghai Jiao Tong University School of Medicine, Shanghai, China; 2 Department of Oral Maxillofacial-Head and Neck Oncology, Ninth People’s Hospital, Shanghai Jiao Tong University School of Medicine, Shanghai, China; 3 CAS Key Laboratory of Molecular Virology & Immunology, Institut Pasteur of Shanghai, Chinese Academy of Sciences, Shanghai, 200031 China

**Keywords:** NLRP3 inflammasome, 5-Fluorouracil, Oral squamous cell carcinoma, Reactive oxygen species, Chemotherapy

## Abstract

**Background:**

5-Fluorouracil (5-FU) is a widely used drug for the therapy of cancer. However, the chemoresistance of tumor cells to 5-FU usually limits its clinical effectiveness. In this study, we explored the role of NLRP3 inflammasome in 5-FU resistance of oral squamous cell carcinoma (OSCC).

**Methods:**

The mRNA and protein expression levels of NLRP3, Caspase1 and IL-1β in resected OSCC specimens or cell lines were measured respectively by quantitative real time-PCR (qRT-PCR) and western blot. NLRP3 and Ki-67 expression in paraffin-embedded OSCC tissues was determined by immunohistochemistry. The correlation between 5-FU treatment and the expression and activation of NLRP3 inflammasome was further examined by evaluating NLRP3 and IL-1β expression in OSCC cell lines without or with NLRP3 knocked down. Cell viabilities of OSCC cells were determined by the MTT assay. Apoptosis and intracellular reactive oxygen species (ROS) of OSCC cells induced by 5-FU were measured by the flow cytometer. The carcinogen-induced tongue squamous carcinoma mice model was established by continuous oral administration of 4-nitroquinoline 1-oxide in wild-type BALB/c, *Nlrp3* ^*−/−*^ and *Caspase1* ^*−/−*^ mice. Tumor incidence were observed and tumor area were evaluated.

**Results:**

In the clinical analysis, expression and activation of NLRP3 inflammasome was clearly increased in OSCC tissues of patients who received 5-FU-based chemotherapy. Multivariate Cox regression analysis revealed that this high expression was significantly correlated with tumor stage and differentiation, and was associated with poor prognosis. Moreover, 5-FU treatment increased expression and activation of NLRP3 inflammasome in OSCC cells in a cell culture system and xenograft mouse model. Silencing of NLRP3 expression significantly inhibited OSCC cell proliferation and enhanced 5-FU-induced apoptosis of OSCC cells. Further investigation showed that intracellular ROS induced by 5-FU promoted the expression and activation of NLRP3 inflammasome and increased the production of interleukin (IL)-1β, which then mediated the chemoresistance. With the carcinogen-induced OSCC model, we found less and later tumor incidence in *Nlrp3* ^*−/−*^ and *Caspase1* ^*−/−*^ mice than wild-type mice. And greater decrease of tumor area was observed in the gene deficient mice treated with 5-FU.

**Conclusions:**

Our findings suggest that NLRP3 inflammasome promoted 5-FU resistance of OSCC both in vitro and in vivo, and targeting the ROS/NLRP3 inflammasome/IL-1β signaling pathway may help 5-FU-based adjuvant chemotherapy of OSCC.

## Background

Head and neck squamous cell carcinoma is the sixth leading cancer by incidence worldwide, and oral squamous cell carcinoma (OSCC) is the most common malignancy of the head and neck [1]. Clinically, although many novel therapeutic approaches have been proposed for this disease, radical surgery combined with adjuvant chemotherapy remains the most effective [2]. However, 5-year survival rate for OSCC patients is still only about 40%–50% [3]. One important reason is chemoresistance [4]. As in many other solid tumors like colon, breast and gastric tumor, 5-fluorouracil (5-FU) is one of the most effective and commonly used drugs for OSCC [5]. Its clinical effectiveness is usually limited because OSCC cells often acquire 5-FU resistance through continuous drug administration [6]. Therefore, it is of great value to explore the molecular mechanisms related to the chemoresistance of OSCC cells, which could ultimately contribute to the establishment of more effective therapeutic strategies.

Nucleotide-binding and oligomerization domain (NOD)-like receptors (NLRs) are a kind of intracellular pattern recognition receptors (PRRs) that can detect microbial components, endogenous stress and damage signals [7, 8]. Among the NLRs family, NLRP3 has been more characterized compared with others. NLRP3 (NLR family, pyrin domain containing 3) together with the adaptor ASC (apoptosis-associated speck-like protein containing a C-terminal caspase-recruitment domain) and caspase-1 can form a protein complex called the NLRP3 inflammasome, which is responsible for the maturation and secretion of proinflammatory cytokines, such as interleukin (IL)-1β and IL-18 etc. [9, 10]. With the exception of its critical role in host defense against a wide range of stress signals like pathogens, environmental stress, metabolic dysregulation and tissue damage [11], aberrant overactivation of NLRP3 inflammasome has been recently identified in various cancers, such as lung and bladder cancer and steroid-resistant acute lymphoblastic leukemia [12–14]. However, the function of NLRP3 inflammasome in OSCC cells has not yet been clearly clarified.

In the current study, we focused on the role of NLRP3 inflammasome in 5-FU chemoresistance of OSCC. We found markedly increased expression of NLRP3 inflammasome in OSCC tissues of patients who received 5-FU-based chemotherapy. We subsequently demonstrated that 5-FU induced expression and activation of NLRP3 inflammasome, which attenuated OSCC cell apoptosis caused by 5-FU. Data from our study imply that targeting the reactive oxygen species (ROS)/NLRP3 inflammasome/IL-1β signaling pathway might be a promising strategy for 5-FU-based chemoresistance.

## Methods

### Patients and tissue specimens

Tissue specimens were obtained from OSCC patients who underwent tumor resection at the Oral and Maxillofacial-Head and Neck Oncology Department of Shanghai Ninth People’s Hospital, Shanghai Jiao Tong University School of Medicine between January 2010 and December 2011. Docetaxel, cisplatin and 5-FU (TPF) induction chemotherapy was administered prior to surgery, and details of the therapeutic regimens had been previously described by Zhong et al [15]. Briefly, TPF induction chemotherapy consisted of docetaxel 75 mg/m^2^ intravenously on day 1, followed by cisplatin 75 mg/m^2^ intravenously on day 1, followed by 5-FU 750 mg/m^2^/day as a 120-h continuous intravenous infusion on days 1 through 5, every 3 weeks for 2 cycles. Surgery was performed at least 2 weeks after completion of induction chemotherapy. The diagnosis and pathological evaluation were based on the guidelines of American Joint Committee on Cancer and were carried out by at least two pathologists. Each patient enrolled in this study gave written informed consent, and all study protocols were approved by the Review Board of Hospital Ethics Committee.

### RNA extraction and quantitative real-time polymerase chain reaction (PCR) (qPCR)

Total RNA was extracted using TRIzol reagent (Invitrogen, San Diego, CA, USA). First-strand cDNA was synthesized from total RNA using the PrimeScript RT Reagent kit (TaKaRa, Shiga, Japan). qPCR was performed by ABI 7900 Real-time PCR System (Life Technologies Corporation, Carlsbad, CA, USA) using the SYBR Premix Ex Taq II (TaKaRa) reaction system. Cycling conditions were configured as follows: initial denaturation (10 min at 95 °C) and then 40 cycles of denaturation (10 s at 95 °C), annealing (15 s at 58 °C), extension (1 min at 72 °C), with a final extension at 72 °C for 5 min. *GAPDH* was used as an endogenous control to normalize for differences in the amount of total RNA in each sample. All quantities were expressed as number of folds relative to the expression of *GAPDH*. The primer sequences were as follows. GAPDH: forward 5’-TGACTTCAACAGCGACACCCA-3’, reverse 5’-CACCCTGTTGCTGT AGCCAAA-3’; NLRP3: forward 5’-CCATCGGCAAGACCAAGA-3’, reverse 5’- ACAGGCTCAGAATGCTCATC-3’; Caspase 1: forward 5’-CAGACAAGGGTGCTG AACAA-3’, reverse 5’-TCGGAATAACGGAGTCAATCA-3’; IL-1β: forward 5’-ATG GCAGAAGTACCTGAGCTC-3’, reverse 5’-TTAGGAAGACACAAATTGCATG-3’.

### Western blot analysis

Tissue protein was extracted using the Radio Immunoprecipitation Assay (RIPA) lysis buffer (Beyotime Biotechnology, Nanjing, China). Equal amounts of protein were electrophoresed on 10% SDS-polyacrylamide gel and then transferred onto polyvinylidene difluoride membranes. The membranes were blocked in 5% fat-free milk with 0.1% Tween-20 for 1 h at room temperature, followed by incubation with different primary antibodies including NLRP3 (1:1000, Sigma, St. Louis, MO, USA), Caspase 1 (1:1000, CST, Danvers, MA, USA), IL-1β (1:1000, Abcam, Cambridge, UK), Caspase 3 (1:1000, CST), cleaved Caspase 3 (1:1000, CST), Bcl-2 (1:800, Proteintech, Rosemont, IL, USA), poly (ADP-ribose) polymerase (PARP) (1:1000, CST), superoxide dismutase (SOD) 2 (1:1000, Abcam), catalase (1:1000, Abcam) or β-actin (1:1000, Sigma) overnight at 4 °C. After washing the membranes for three times, the blots were incubated with fluorescent-based anti-rabbit IgG secondary antibody (1:1000, Fermentas, Vilnius, Lithuania) for 1 h at room temperature. Protein was visualized using Odyssey Infrared Imaging System (LI-COR Biosciences, Lincoln, NE, USA).

### Immunohistochemistry

Immunohistochemistry was performed as previously described [16]. Formalin-fixed, paraffin-embedded samples of OSCC tissues were consecutively cut into 5-μm-thick sections. Immunostaining was performed using the primary antibodies against NLRP3 (1:400, Sigma), IL-1β (1:200, Abcam) or Ki-67 (1:400, CST) and detected by anti-mouse or anti-rabbit GTVision Two-step Visualization System (Genetech, Shanghai, China). Tissue sections were counterstained with Mayer’s hematoxylin. For NLRP3 and IL-1β, the staining intensity score (IS) was classified as zero (negative), one (weak), two (moderate) and three (strong). Staining proportion score (PS) was classified as 0 (0%), 1 (1%–25%), 2 (26%–50%), 3 (51%–75%), and 4 (76%–100%). The final staining score was calculated by multiplying IS by PS. According to the final score, patients with different NLRP3 expression were divided into two groups: low expression group (0–6) and high expression group (7–12). For Ki-67, patients were also grouped by different expression: low expression group (nuclear staining tumor cells <46%) and high expression group (nuclear staining tumor cells ≥46%).

### Cell lines and reagents

The human OSCC cell line CAL-27 was obtained from the American Type Culture Collection (Manassas, VA, USA), and WSU-HN6 was obtained from the National Institutes of Health (Bethesda, MD, USA). All these cells were maintained in Dulbecco’s modified Eagle’s medium supplemented with 10% fetal bovine serum (Gibco, New York, NY, USA) and cultured in a humidified atmosphere of 5% CO_2_ at 37 °C. 5-FU, 4-NQO and recombinate human IL-1β were purchased from Sigma. NAC was from Sangon Biotech (Shanghai, China).

### NLRP3 shRNA interference and transfection

Commercially available NLRP3 shRNA constructs were obtained from Genechem (Shanghai, China) and were used to knock down NLRP3 expression in CAL27 and WSU-HN6 cells. The NLRP3 shRNA duplex sequences are as follows: forward 5’-GATCCAGCCAACAGGAGAACTTTCCTTCCTGTCAGAGAAAGTTCTCCTG TTGGCTTTTTTG-3’, reverse 5’-AATTCAAAAAAGCCAACAGGAGAACTTTCT CTGACAGGAAGGAAAGTTCTCCTGTTGGCTG-3’. A control vector (shcontrol) was constructed (5’-GAAGCAGCACGACTTCTTC-3’) with no significant homology to any mammalian gene sequence. CAL27 and WSU-HN6 cells were transfected with 5 × 10^5^ transfecting units/mL of lentivirus particles for 12 h, and puromycin was used to select the stable transfected cells for another 7 days.

### Cell viability assay

Cell viability was measured by 3-(4,5-dimethylthiazol-2-yl)-2,5-diphenyltetrazolium bromide (MTT) assay. OSCC cells (5 × 10^3^ cells/well) were seeded in 96-well plates and cultured for 24 h. Then cells were exposed to 5-FU, and cells in the control group were treated with the same volume of culture medium. After treatment, a 20-μl aliquot of MTT solution [0.5 mg/ml in phosphate-buffered saline (PBS); Sigma] was supplemented into each well for an additional 4 h incubation. The supernatant was then discarded and 150 μl dimethyl sulfoxide was added to dissolve the formazan crystals. Optical density was read at 490 nm on a microplate reader (Bio-Rad, Hercules, CA, USA).

### Flow cytometry analysis of apoptosis and intracellular ROS

The apoptosis of tumor cells was analyzed using the Annexin V Apoptosis Detection Kit (BD Biosciences, San Diego, CA, USA). Tumor cells were harvested and washed by PBS, resuspended in prediluted binding buffer, and stained with annexin V–APC for 30 min at room temperature. After being washed, the cells were immediately subjected to apoptosis analyses by flow cytometry. For the detection of intracellular ROS, cells were incubated with serum-free culture medium containing 10 μM DCFH-DA (Beyotime Biotechnology) for 20 min at 37 °C, then washed twice with serum-free culture medium to remove extracellular DCFH-DA. The cells were resuspended in PBS and subjected to flow cytometric analysis. All flow cytometry was performed on the FACS Calibur Flow Cytometer (BD Biosciences) and the data were analyzed by FlowJo software (Treestar, Ashland, OR, USA).

### Establishment of the OSCC xenograft nude mouse model

Four-week old male BALB/c nude mice were purchased from the Shanghai Laboratory Animal Center (Shanghai, China). All mice were housed under pathogen-free conditions in the animal care facilities of Shanghai Ninth People’s Hospital, Shanghai Jiao Tong University School of Medicine. WSU-HN6 or CAL27 cells (5 × 10^6^) were injected subcutaneously into the back next to the limb of nude mice. When the average tumor volume reached ~50 mm^3^, 5-FU was administrated intraperitoneally with a dose of 20 mg/kg every 4 days for 3 weeks. Tumors were measured with Vernier calipers and tumor volumes were calculated according to the following formula: length × width^2^/2. At the end of the experiments, all the mice were killed and tumors were obtained and weighed. The animal welfare and experimental procedures were carried out strictly in accordance with the Guide for the Care and Use of Laboratory Animals (The Ministry of Science and Technology of China, 2006) and the related ethical regulations of the hospital. All efforts were made to minimize animal suffering and to reduce the number of animals used. All experimental procedures received approval by the Laboratory Animal Care and Use Committees of the hospital.

### Mouse model for 4-NQO-induced oral cancer

*NLRP3*-deficient (*Nlrp3* ^*−/−*^) and *Caspase1*-deficient (*Caspase1* ^*−/−*^) mice initially on C57BL/6 genetic background had been described before [17, 18]. These mice were crossed onto BALB/c background for 9 generations for the current study. Wild-type male BALB/c mice (6 weeks old,) were purchased from Shanghai Laboratory Animal Center. All animals were housed under pathogen-free conditions in the animal care facilities of Shanghai Ninth People’s Hospital. The carcinogen-induced tongue squamous carcinoma mouse model was set up as previously described by Tang et al. with some modification [19]. 4-nitroquinoline 1-oxide (4-NQO) powder was dissolved in propylene glycol as a stock solution and was diluted in drinking water to a final concentration of 150 μg/ml. After 18 weeks of induction, mice were randomly divided into two groups. One group was used to assess tumor incidence and area. Mice in another group were injected with 50 mg/kg 5-FU intraperitoneally twice weekly. After 4 weeks, tumor area was drawn and measured by the tool of freehand sections in the ImageJ software (National Institutes of Health). The details of the technical method were referred as previously described [20]. The decrease in tumor area in *Nlrp3* ^*−/−*^ and *Caspase1* ^*−/−*^ mice was compared with that of wild-type mice. All of the animal procedures were conducted in accordance with the Shanghai Ninth People’s Hospital, Shanghai Jiao Tong University School of Medicine Animal Care guidelines. All efforts were made to minimize animal suffering.

### Statistical analysis

Data are shown as mean ± SD. The two-tailed Student’s *t* test was used to determine the statistical significance of the differences between the two groups. The *χ* ^*2*^ or Fisher’s exact tests were used to evaluate the relation between NLRP3 and Ki-67 expression and the clinicopathological parameters of the patients. The overall survival (OS) and disease-free survival (DFS) rates were calculated using the Kaplan-Meier method, and the differences were calculated by the log-rank test. The Cox proportional hazards model was used to evaluate the influence of various clinicopathological factors and NLRP3 expression on the survival of OSCC patients. *P* < 0.05 was considered statistically significant. All statistical analyses were performed using SPSS version 19.0 software (SPSS Inc., New York, NY, USA).

## Results

### Aberrant increased expression and activation of NLRP3 inflammasome in OSCC tissues of 5-FU-treated patients

**Fig. 1:**
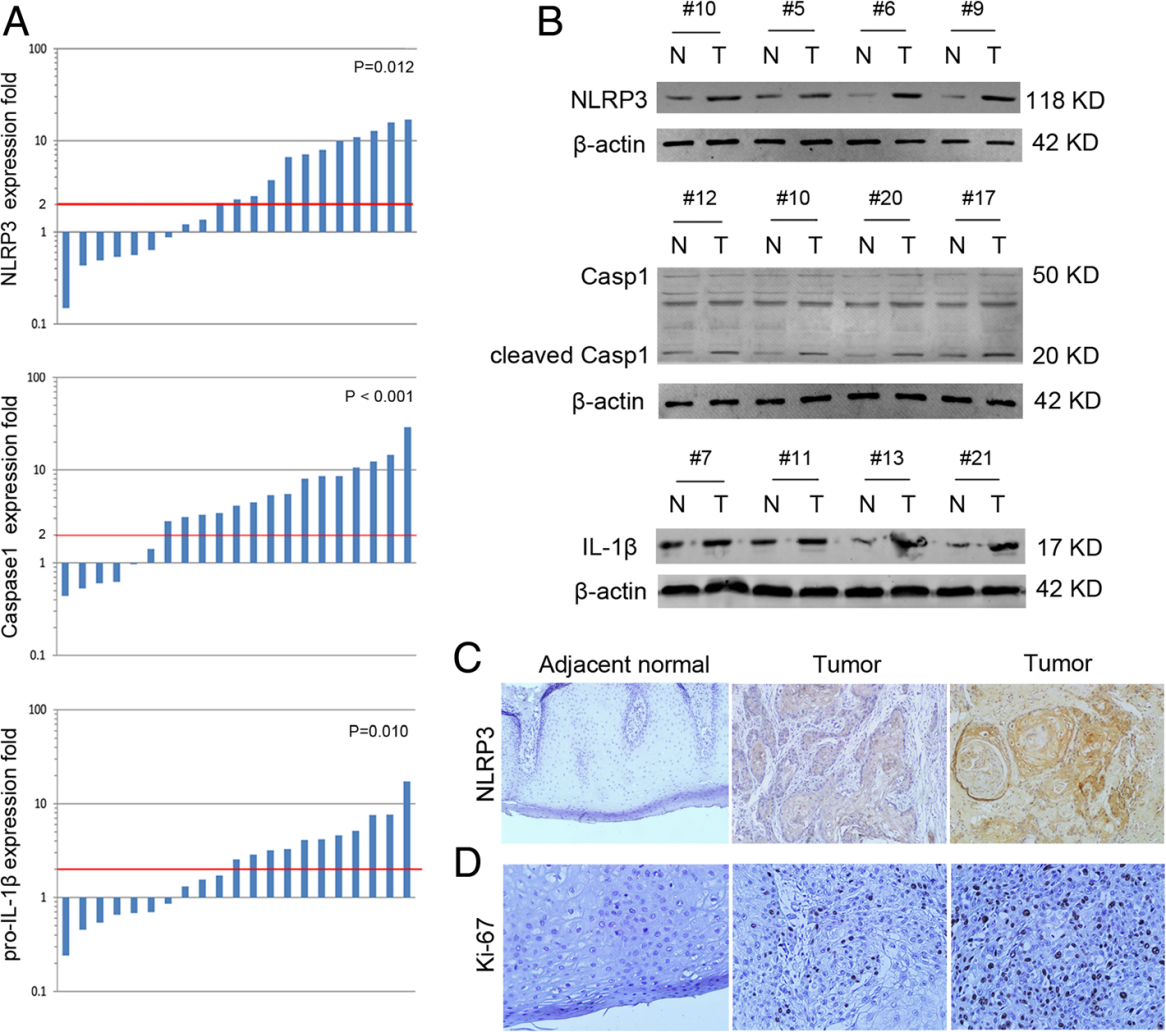
NLRP3 inflammasome expression is increased in OSCC tissues. **a** mRNA expression of NLRP3, Caspase1 and pro-IL-1β in 21 paired OSCC and adjacent normal tissues was determined by real-time qPCR. **b** Protein expression of NLRP3, Caspase1 and IL-1β in 21 paired OSCC and adjacent normal tissues by western blot. **c** Immunohistochemical staining for NLRP3 and Ki-67. (magnification 100× for NLRP3 staining and 200× for Ki-67 staining)

Twenty-one randomly selected, paired OSCC cases that received 5-FU-based chemotherapy before radical surgery were used to evaluate the expression of NLRP3, Caspase1 and IL-1β. There was a ≥2-fold increase in the mRNA levels of NLRP3 (12/21, 57.1%, *P* = 0.012), Caspase1 (15/21, 71.4%, *P* < 0.001) and pro-IL-1β (11/21, 52.4%, *P* = 0.010) in OSCC tissues compared with adjacent noncancerous tissues (Fig. 1a). As cleaved Caspase1 is an established indicator of NLRP3 inflammasome activation [10], we measured cleaved Caspase1 in subsequent western blot analysis. The results confirmed high protein levels of NLRP3, cleaved Caspase1 and IL-1β in OSCC tissues from 5-FU-treated patients (Fig. 1b).

To further determine the clinicopathological significance of NLRP3 inflammasome, immunohistochemistry was performed in 103 OSCC specimens after 5-FU-based chemotherapy. Only 32 (31.1%) showed low NLRP3 expression, while 71 (68.9%) showed high NLRP3 expression (Fig. 1c, Table 1). Upregulated expression of NLRP3 was significantly associated with T stage (*P* = 0.001) and tumor differentiation (*P* = 0.010) of OSCC. Ki-67 is one of the most commonly used proliferation markers of tumor cells, thus we examined its expression and evaluated its relation with NLRP3. We found that high expression of Ki-67 was significantly associated with T stage (*P* = 0.013), N stage (*P* = 0.006) and tumor differentiation (*P* = 0.018) of OSCC (Fig. 1d, Table 1). And a positive correlation between Ki-67 and NLRP3 was identified by Spearman’s correlation coefficient test (*P* = 0.014, Table 2). These data indicate that NLRP3 inflammasome is commonly involved in OSCC development after 5-FU-based chemotherapy.

**Table 1:** Correlation between NLRP3 expression and clinicopathological features in patients with OSCC (*n* = 103)

Variable	Number	NLRP3 expression		*P* value*	Ki-67 expression		*P* value*
		Low (32)	High (71)		Low (38)	High (65)	
Smoking status**							
Current/former	51	18	33	0.359	20	31	0.629
Never	52	14	38		18	34	
Alcohol intake***							
Yes	42	12	30	0.650	15	27	0.837
No	61	20	41		23	38	
Age							
< 60	59	20	39	0.472	22	37	0.923
≥ 60	44	12	32		16	28	
Gender							
Male	63	19	44	0.802	26	37	0.298
Female	30	13	27		12	28	
Location							
Tongue	37	12	25	0.976	10	27	0.228
Buccal	24	8	16		10	14	
Gingiva	16	5	11		8	8	
Mouth floor	13	3	10		3	10	
Other	13	4	9		7	6	
T stage							
T1 + T2	45	22	23	0.001*	23	22	0.013*
T3 + T4	58	10	48		15	43	
N stage							
N0	47	16	31	0.550	24	23	0.006*
N1 + N2	56	16	40		14	42	
M stage							
M0	102	32	70	0.500	39	63	0.433
M1	1	0	1		0	1	
Differentiation							
Well	39	18	21	0.010*	20	19	0.018*
Moderate + Poor	64	14	50		18	46	

*P-values are based on chi-squared or Fisher’s exact test. *P* < 0.05 indicates a significant association among the variables

**Former/current smokers defined as at least a one pack-year history of smoking

***Alcohol intake was defined as current alcohol intake of more than one drink per day for 1 year. Others were classified as no alcohol intake

**Table 2:** The association between NLRP3 and Ki67 expression

	Ki-67 expression		*P* value	*r*
	Low	High		
NLRP3 Low expression	18	14	0.006*	0.269
NLRP3 High expression	20	51		

*P-values are based on Spearman’s correlation coefficient test. *P* < 0.05 indicates a significant association among the variables

### High NLRP3 expression is associated with poor clinical outcome in 5-FU-treated OSCC patients

**Fig. 2:**
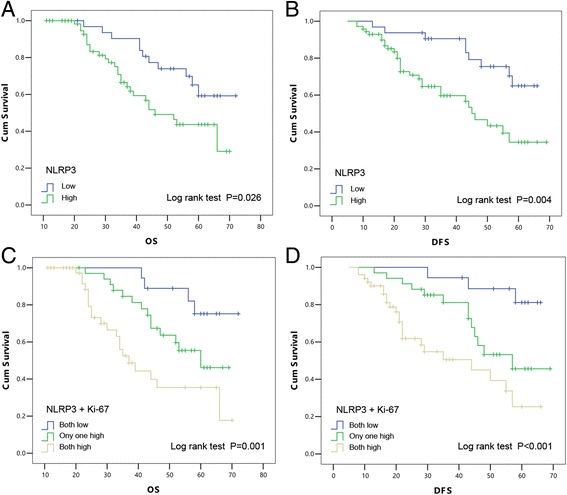
Kaplan–Meier with log rank test analysis of the OS and DFS rates for 103 OSCC patients. The influence of NLRP3 expression on OS (**a**) and DFS (**b**) of OSCC patients. Combined assessment of the influence of NLRP3 and Ki-67 expression on OS (**c**) and DFS (**d**) of OSCC patients

To assess the potential association between NLRP3 expression and the survival of OSCC patients, Kaplan-Meier analysis with a log rank test for 5-year OS and DFS was performed. Patients with high NLRP3 expression usually had poorer OS and DFS than those with low NLRP3 expression (*P* = 0.026 and *P* = 0.004, respectively, Fig. 2a and b). Furthermore, with regard to the concomitant expression of NLRP3 and Ki-67, we divided the patients into three groups: Group 1, patients with high expression of NLRP3 and Ki-67; Group 2, patients with high expression of only one of them; and Group 3, patients with low expression of NLRP3 and Ki-67. Notably, patients in Group 2 had significantly poorer OS and DFS than those in Group 3, and patients in Group 1 had the worst OS and DFS (*P* = 0.001 and *P* < 0.001, respectively, Fig. 2c and d). Using a multivariate Cox regression analysis, we found that positive expression of NLRP3 was a significant independent prognostic factor for OS [hazard ratio (HR) 3.81, 95% confidence interval (CI) 0.36–8.08, *P* = 0.014, Table 2]. Expression of NLRP3 combined with Ki-67 was a significant independent prognostic factor for OS and DFS (OS: HR 7.79, 95% CI 3.48–13.27, *P* < 0.001, DFS: HR 12.27, 95% CI 4.25–19.45, *P* < 0.001, Table 3). Collectively, these results suggest that NLRP3 has clinical significance in the diagnosis and prognosis of OSCC patients receiving 5-FU-based chemotherapy.

**Table 3:** Multivariate Cox proportional hazard models for overall survival (OS) and disease-free survival (DFS)

Variable	Overall survival (OS)		Disease-free survival (DFS)	
	HR (95% CI)	*P* value	HR (95% CI)	*P* value
T stage (T1 + T2 vs. T3 + T4)	1.73 (1.09–4.01)	0.221	1.43 (0.35–5.52)	0.136
N stage (N0 vs. N1 + N2)	5.95 (3.62–9.22)	<0.001*	3.67 (1.59–7.44)	0.018*
M stage (N0 vs. M1)	4.04 (1.79–12.55)	0.014*	5.43 (1.69–20.04)	<0.001*
Differentiation (Well vs. Moderate + Poor)	2.99 (1.30–6.88)	0.025*	2.21 (1.05–9.62)	0.029*
NLRP3 (Low vs. High)	3.81 (0.36–8.08)	0.014*	1.88 (0.33–4.12)	0.194
NLRP3/Ki-67 (Both low vs. Both high)	7.79 (3.48–13.27)	<0.001*	12.27 (4.25–19.45)	<0.001*

*HR* hazard ratio, *CI* confidence interval; *P*-values are based on Likelihood Ratio test; *P < 0.05 indicates significant difference

### 5-FU induces expression and activation of NLRP3 inflammasome in OSCC cells

**Fig. 3:**
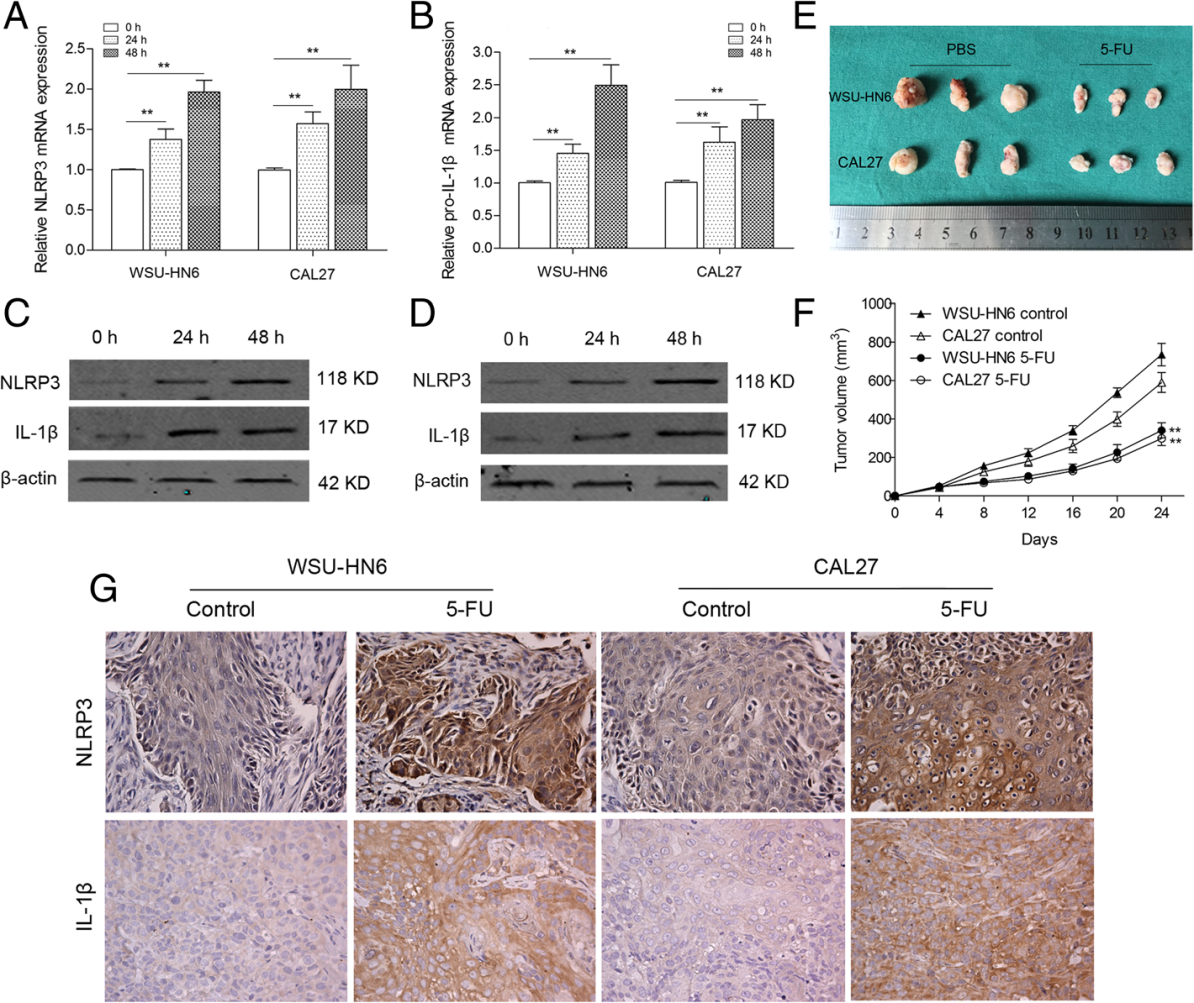
5-FU treatment induced expression of NLRP3 and IL-1β in vitro and in vivo. WSU-HN6 and CAL27 cells were treated with 10 μM 5-FU for 0, 24 and 48 h. mRNA expression of NLRP3 (**a**) and pro-IL-1β (**b**) was quantified by qPCR. Protein levels of NLRP3 (**c**) and IL-1β (**d**) in both cells were determined by western blot. (**e**) Representative image of tumors from OSCC xenografted nude mice with or without 5-FU treatment. **f** Tumor volumes of mice treated with or without 5-FU. **g** Immunohistochemistry indicated highly elevated expression of NLRP3 and IL-1β in tumors of 5-FU-treated mice (original magnification 200×). Three mice were included for each group, and the results are representative of three experiments (**P < 0.01)

To investigate the correlation between NLRP3 inflammasome and chemotherapeutic response, we treated the OSCC cell lines-WSU-HN6 and CAL27 with 5-FU. Real-time PCR showed that mRNA level of NLRP3 in WSU-HN6 and CAL27 cells was elevated in a time-dependent manner (Fig. 3a). We also examined pro-IL-1β mRNA expression and similar results were obtained (Fig. 3b). Western blot analysis confirmed the increase of NLRP3 and IL-1β expression at protein level in WSU-HN6 and CAL27 cells following 5-FU treatment (Fig. 3c and d). This phenomenon was also observed in an OSCC xenograft mouse model. We established the OSCC xenograft mouse model with WSU-HN6 and CAL27 cells and found that 5-FU treatment significantly limited OSCC growth and obviously increased the expression of NLRP3 and IL-1β in tumor cells (Fig. 3e–g). Thus, 5-FU induces the expression and activation of NLRP3 inflammasome in OSCC cells both in vitro and in vivo.

### NLRP3 knockdown enhances the decrease in viability of OSCC cells induced by 5-FU

**Fig. 4:**
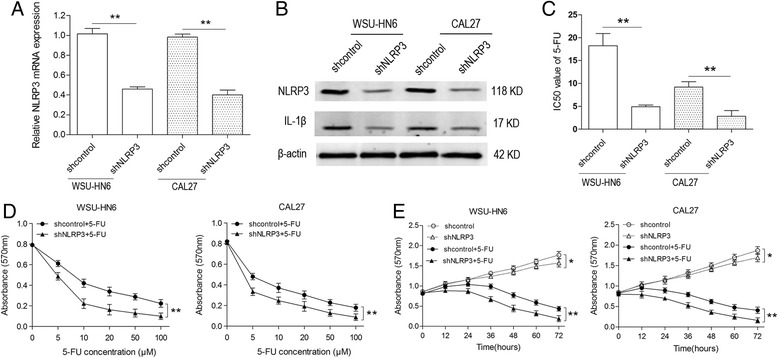
NLRP3 knockdown decreases OSCC cell viability. WSU-HN6 and CAL27 OSCC cell lines were transfected with NLRP3 or control shRNA. Total RNA and protein were obtained. **a** mRNA expression of NLRP3 was determined by qPCR. **b** Protein expression of NLRP3 and IL-1β was determined by western blot. **c** IC50 values of 5-FU in WSU-HN6 and CAL27 cells were examined using the MTT assay. **d** Viability of shcontrol and shNLRP3 OSCC cell lines with various concentrations of 5-FU treatment (0 μM, 5 μM, 10 μM, 20 μM, 50 μM and 100 μM) for 48 h was determined. **e** Viability of shcontrol and shNLRP3 OSCC cell lines with/without 5-FU treatment (5 μM) was detected at different time points (0 h, 12 h, 24 h, 36 h, 48 h, 60 h and 72 h). Data are representative of three experiments (*P < 0.05, **P < 0.01)

To further explore the role of NLRP3 inflammasome in 5-FU-treated OSCC cells, NLRP3 expression was knocked down. WSU-HN6 and CAL27 cells were stably transfected with NLRP3 short hairpin (sh) RNA to silence the expression of NLRP3. Real-time PCR and western blot were used to confirm the depletion of NLRP3 at mRNA and protein level respectively (Fig. 4a and b). Decreased expression of IL-1β confirmed the reduced expression and activation of NLRP3 inflammasome (Fig. 4b). With the MTT assay, we found that NLRP3 knockdown reduced the IC50 of 5-FU in OSCC cells and synergistically enhanced the effect of 5-FU in a concentration- and time-dependent manner (Fig. 4c–e). Therefore, these data imply that NLRP3 inflammasome is involved in 5-FU chemoresistance of OSCC cells.

### NLRP3 knockdown increases 5-FU-induced apoptosis of OSCC cells

**Fig. 5:**
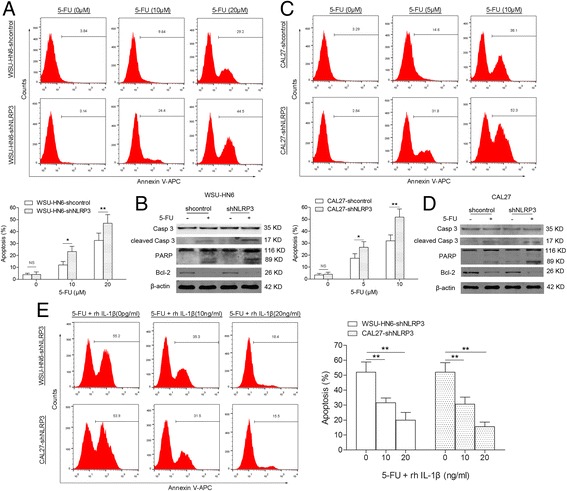
NLRP3 knockdown sensitizes OSCC cells to apoptosis. WSU-HN6 and CAL27 cells were transfected with NLRP3 shRNA or control shRNA. Apoptosis of WSU-HN6 (**a**) and CAL27 (**c**) cells with or without 5-FU treatment was determined by FACS. Apoptosis-related protein was determined by western blot (**b** and **d**). **e** rIL-1β administration reversed the increase of 5-FU-induced apoptosis in WSU-HN6 and CAL27 cells with NLRP3 knockdown. Results are representative of three experiments (*P < 0.05, **P < 0.01)

Then we assessed the role of NLRP3 inflammasome in 5-FU-induced apoptosis of OSCC cells. Results showed that 5-FU induced the apoptosis of OSCC cells in a concentration-dependent manner, and knockdown of NLRP3 by shNLRP3 enhanced the proapoptotic effect of 5-FU (Fig. 5a and c). To elucidate the molecular mechanisms, related apoptotic and antiapoptotic proteins were determined. Compared with that of 5-FU-treated shcontrol OSCC cells, higher expression of cleaved Caspase 3 and PARP was observed in 5-FU-treated shNLRP3 OSCC cells. Lower expression of the antiapoptotic protein Bcl-2 was found in shNLRP3 OSCC cells (Fig. 5b and d). To confirm the role of activated NLRP3 inflammasome, a rescue experiment with IL-1β was performed, in which 10 or 20 ng/ml recombinant human IL-1β (rIL-1β) was administered to the shNLRP3 OSCC cells together with 5-FU. rIL-1β compromised the increased apoptosis in shNLRP3 OSCC cells (Fig. 5e). Overall, the increased expression and activation of NLRP3 inflammasome in OSCC cells induced by 5-FU decreases the proapoptotic effect of 5-FU.

### 5-FU-induced intracellular ROS promote NLRP3 inflammasome expression and activation

**Fig. 6:**
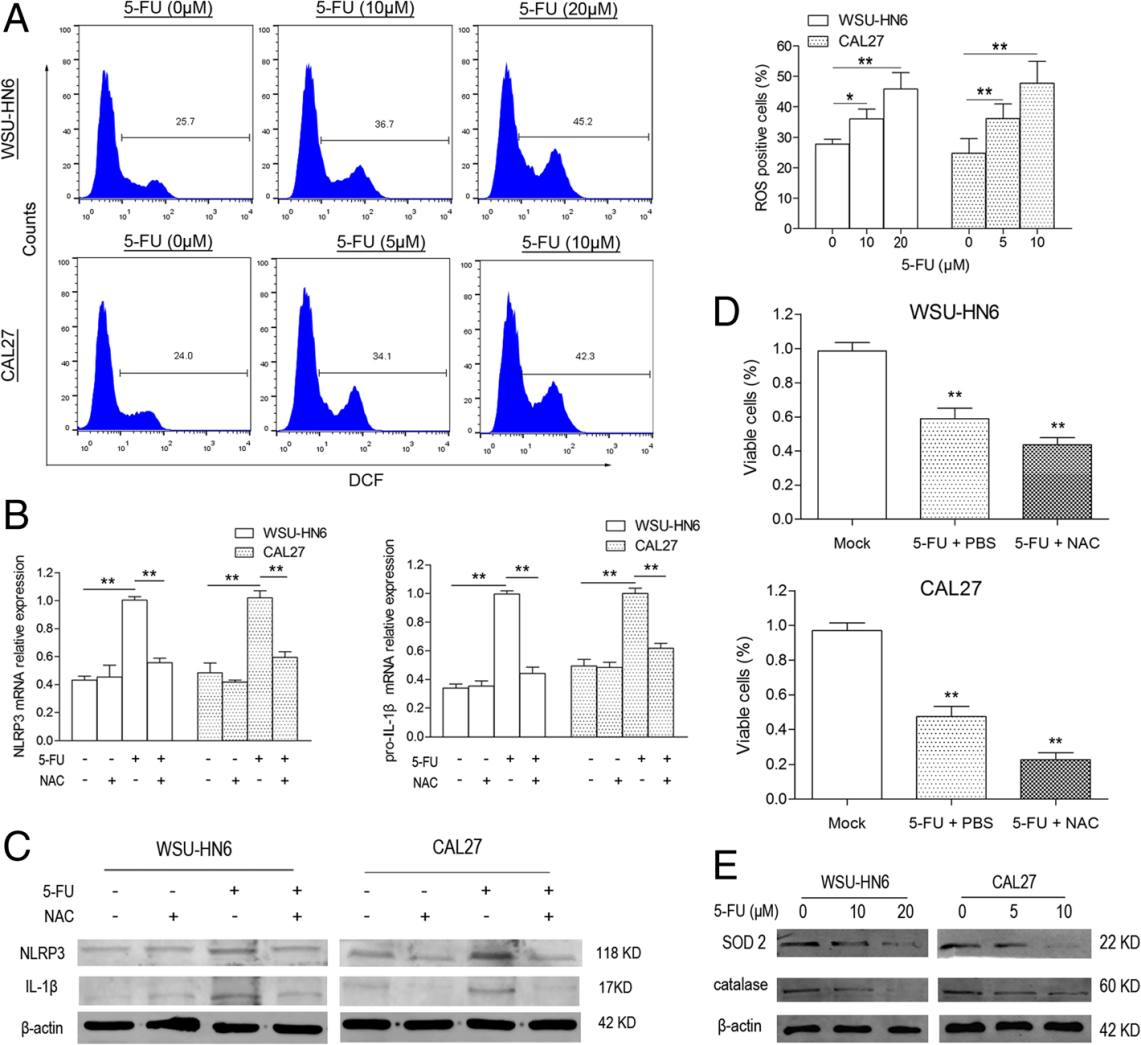
5-FU-induced ROS activates NLRP3 inflammasome in OSCC cells. **a** Intracellular ROS production after 5-FU treatment was detected using DCFH-DA staining in WSU-HN6 and CAL27 cells. mRNA (**b**) and protein (**c**) expression of NLRP3 and IL-1β in WSU-HN6 and CAL27 cells treated with 5-FU and/or NAC (5 mM) was detected by qPCR or western blot. **d** The percentage of viable cells with 5-FU and/or NAC treatment was determined using trypan blue. **e** SOD2 and catalase expression in WSU-HN6 and CAL27 cells treated with or without 5-FU was detected. Data are representative of three experiments (*P < 0.05, **P < 0.01)

To clarify the mechanism of expression and activation of NLRP3 inflammasome in OSCC cells treated with 5-FU, intracellular ROS levels were determined. 5-FU increased ROS production as well as expression and activation of NLRP3 inflammasome in OSCC cells, while *N*-acetylcysteine (NAC), an antioxidant that suppressed intracellular ROS, reversed the effect of 5-FU on NLRP3 inflammasome (Fig. 6a–c). And NAC treatment significantly enhanced 5-FU-induced apoptosis (Fig. 6d). We then went on to detect the antioxidant enzyme levels. Two main antioxidant enzymes, SOD2 and catalase, were suppressed in WSU-HN6 and CAL27 cells by 5-FU treatment in a dose-dependent manner (Fig. 6e). Therefore, by inhibiting the expression of antioxidant enzymes, 5-FU increases intracellular ROS, which lead to the expression and activation of NLRP3 inflammasome and finally induce the 5-FU-chemoresistance of OSCC cells.

### Deficiency of NLRP3 inflammasome strengthens the antitumor effect of 5-FU in vivo

**Fig. 7:**
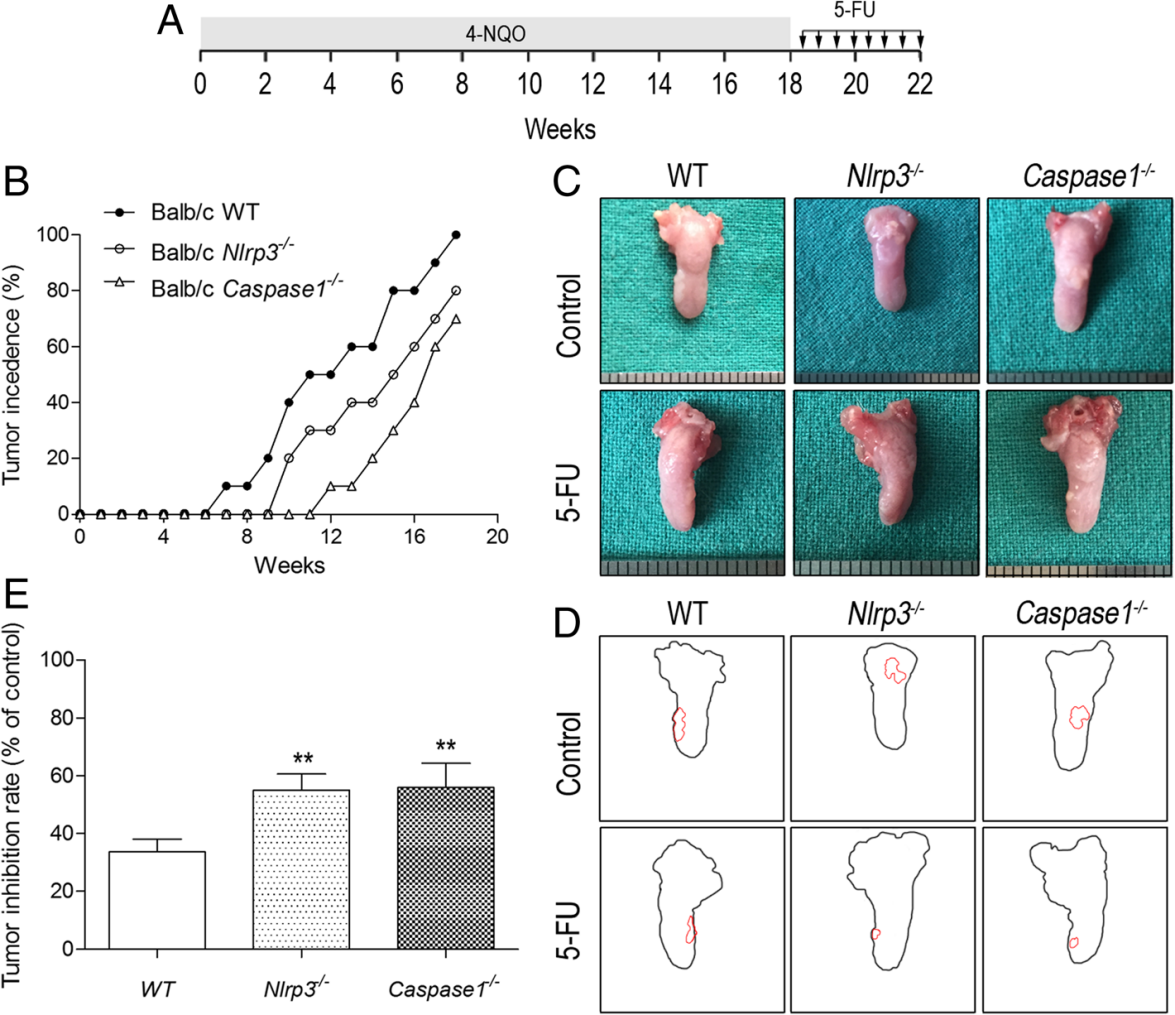
NLRP3 inflammasome promotes OSCC growth and restrains the antitumor effect of 5-FU in vivo. **a** Schematic representation for the establishment of 4-NQO-induced oral cancer mouse model and 5-FU treatment regimens. **b** Tumor incidence in wild-type, *Nlrp3* ^*−/−*^ and *Caspase1* ^*−/−*^ mice. **c** Representative photographed pictures of tongues with tumors from mice treated with or without 5-FU. **d** Digital outline of each representative tongue from control and 5-FU treated mice as described in panel C. Red lining indicated tumor burden in the respective tongue. **e** Tumor areas as indicated in 7B were quantified by ImageJ software in arbitrary units. The inhibition rate of tumor area in wild type, *Nlrp3* ^*−/−*^ and *Caspase1* ^*−/−*^ mice after 4 weeks of 5-FU treatment. Five mice were included in each group, and results are representative of three experiments (*P < 0.05, **P < 0.01)

To further verify the chemoresistant effect of NLRP3 inflammasome in vivo, we established a tongue squamous carcinoma model by 4-NQO in wild-type BALB/c, *Nlrp3* ^*−/−*^ and *Caspase1* ^*−/−*^ mice [19]. The experimental scheme is shown in Fig. 7a. 4-NQO was added to the drinking water of all mice for 18 weeks. With OSCC formation in the tongue at the 18th week, mice were injected with 5-FU twice weekly for 4 weeks and the reduction in tumor area was evaluated. *Nlrp3* ^*−/−*^ and *Caspase1* ^*−/−*^ mice showed later occurrence of tongue carcinoma and smaller tumor area than wild-type mice (Fig. 7b). And 5-FU significantly suppressed tumor growth in all three groups of mice, with greater inhibition in *Nlrp3* ^*−/−*^ and *Caspase1* ^*−/−*^ mice than in wild-type mice (Fig. 7c–e). Hence, NLRP3 inflammasome indeed involves in 5-FU chemoresistance of OSCC, and deficiency of NLRP3 inflammasome could strengthen the antitumor effect of 5-FU.

## Discussion

In this study, we demonstrate for the first time that NLRP3 inflammasome plays an important role in 5-FU-based chemoresistance of OSCC. Our data support the hypothesis that activation of NLRP3 inflammasome could serve as a driver of OSCC development in patients receiving preoperative 5-FU-based adjuvant chemotherapy. Furthermore, our results indicate that lower NLRP3 expression is related to a less aggressive OSCC phenotype, and decreased NLRP3 inflammasome activation strengthens the antitumor effect of 5-FU in OSCC cells. We also find that increased intracellular ROS induced by 5-FU is a key regulator of NLRP3 inflammasome expression and activation in OSCC cells. Collectively, these results indicate that high expression and activation of NLRP3 inflammasome might confer a more malignant phenotype of OSCC, and regulate the chemoresistance of OSCC cells to 5-FU-based therapy.

It has been acknowledged that chronic inflammation is an important factor in carcinogenesis and tumor progression [21, 22], and cancer-related inflammation has been recognized as the seventh hallmark of cancer [23]. Tumor incidence and development are determined by oncogene and tumor suppressor gene aberrations as well as tumor microenvironment [24]. Cytokines and chemokines in the tumor microenvironment were once considered to be released mainly by immune cells, however, many studies have demonstrated that tumor cells themselves also produce these inflammatory mediators [25, 26]. Abnormal expression and activation of PRRs in tumor cells play critical roles in this process. Mastorci et al. have reported that mantle cell lymphoma (MCL) cell lines and primary MCL cells express high levels of Toll-like receptor (TLR) 2 and TLR5, which increases IL-4 and IL-6 production and promotes the development of MCL [27]. Schwartz et al. have found that TLR3 is constitutively expressed in human pancreatic cancer and melanoma cells, and inhibition of TLR signaling reduces interferon-β, IL-6 and chemokine CXC ligand 10 production, which results in decreased proliferation and migration of tumor cells [28]. Mehmeti et al. have shown a strong correlation between expression of TLR4 and proinflammatory mediators like IL-6 and IL-8 in primary breast cancer, and TLR4 protein expression is correlated with decreased survival [29]. Similarly, our previous study revealed an important role of TLR4 in OSCC [30]. We found that TLR4 and myeloid differentiation primary response gene 88 (MyD88) were highly expressed in OSCC cells and activation of TLR4 induced production of IL-6, IL-8 and vascular endothelial growth factor. OSCC cells were also found to be resistant to cisplatin-mediated apoptosis after lipopolysaccharide treatment. In gastric cancer, it has been shown that the TLR/MyD88 pathway is needed for the inflammatory response in tumor tissues, and plays a role in maintenance of stemness in gastric tumor cells [31]. Besides TLRs, other PRRs/NLRs show aberrant function and expression in tumors [32, 33]. To date, 22 human and 34 mouse NLRs have been identified, including NLRP1, NLRP3, NLRP6 and NLRC4 etc. [34]. NLRP3, as part of the inflammasome protein complex, is better characterized in cancer compared with the others. Tarassishin et al. found that increased IL-1β processing by upregulated NLRP3 expression in human astrocytes and astrogliomas conferred them a mesenchymal phenotype, including increased migratory capacity, unique gene signature and proinflammatory signaling [35]. Chow et al. showed that NLRP3-inflammasome-deficient mice had reduced incidence of methylcholanthrene-induced fibrosarcoma, and deficiency of NLRP3 contributed to decreased tumor metastasis via increased activation of natural killer cells [36]. Li et al. have demonstrated that the NLRP3 gene signature may also serve as a predictive biomarker for glioma patients [37]. However, its clinical significance and potential role in OSCC remains unknown. In the present study, we confirmed that NLRP3 inflammasome involved in OSCC development after 5-FU-based chemotherapy, and patients with higher NLRP3 expression had poorer survival than those with lower NLRP3 expression.

5-FU is an antimetabolite chemotherapeutic agent that is widely used for the treatment of cancer [38]. However, 5-FU resistance with different causes threatens the clinical outcome of OSCC patients, and the exact mechanism is unclear [39]. Recently, the role of NLRP3 inflammasome in this process has aroused the interests of researchers. Bruchard et al. found that increased activation of NLRP3 inflammasome in myeloid-derived suppressor cells (MDSCs) by 5-FU limited its antitumor efficacy [40]. Ghiringhelli et al. showed that the 5-FU-derived activation of NLRP3 inflammasome in MDSCs promoted tumor angiogenesis by eliciting a T helper 17 response [41]. In this study, we found that expression of NLRP3 and IL-1β was significantly upregulated in WSU-HN6 and CAL27 cells following 5-FU treatment. We also found that downregulating NLRP3 expression significantly enhanced the decrease of cell viability caused by 5-FU. These findings were confirmed in *Nlrp3* ^*−/−*^ and *Caspase1* ^*−/−*^ mice. With the 4-NQO-induced tongue squamous carcinoma model (a widely used OSCC model), we demonstrated that deficiency of NLRP3 inflammasome enhanced the anti-tumor effect of 5-FU.

Some recent investigations have recognized a growing number of diverse stimuli involved in NLRP3 inflammasome activation, and ROS is the most important one [42, 43]. Increased ROS stress has been observed in a wide spectrum of human cancers and is associated with oncogenic signals such as c-myc and Ras [44, 45]. Suzuki et al. found that treatment of human pancreatic cancer stem cells with 5-FU increased the intracellular ROS [46]. Similarly, Darsigny et al. found that 5-FU treatment led to efficient production of intracellular ROS in colorectal cancer cells [47]. Given that 5-FU induces intracellular ROS production in many cancer cells, we speculated whether 5-FU would increase ROS in OSCC cells and activate NLRP3 inflammasome. Our results demonstrated that intracellular ROS levels were indeed upregulated in 5-FU-treated OSCC cells, and later activated NLRP3 inflammasome, which promoted IL-1β production and 5-FU chemoresistance. When we treated OSCC cells with NAC, an antioxidant that suppressed intracellular ROS, the effect of 5-FU on NLRP3 inflammasome was reversed. However, the mechanisms of chemoresitance to 5-FU treatment lie on multiple aspects. For example, Zhang et al have demonstrated that tumor associated macrophages (TAMs) became activated during treatment with 5-FU and secreted putrescine that protected the colorectal cancer cells against chemotherapy with 5-FU by attenuating JNK-caspase-3 pathway-mediated cell apoptosis [48]. Therefore, whether a similar work mechanism existed in TAMs of OSCC required needs further study.

## Conclusions

Our results demonstrated that aberrant expression and activation of NLRP3 inflammasome contributed to 5-FU chemoresistance of OSCC cells. Intracellular ROS was increased by 5-FU and subsequently activated the NLRP3 inflammasome in OSCC cells. Thus, NLRP3 inflammasome might be used to identify patients who are more likely to benefit from 5-FU-based chemotherapy, and targeting the ROS/NLRP3 inflammasome/IL-1β signaling pathway may help the therapy of 5-FU-resistant OSCC.

## Abbreviations

4-NQO: 4-nitroquinoline 1-oxide
5-FU: 5-Fluorouracil
ASC: Apoptosis-associated speck-like protein containing a C-terminal caspase-recruitment domain
CI: Confidence interval
DFS: Disease-free survival
HR: Hazard ratio
MDSC: Myeloid-derived suppressor cell
MTT: 3-(4, 5-dimethylthiazol-2-yl)-2, 5- diphenyltetrazolium bromide
NAC: *N*-acetylcysteine
NLRP3: NLR family, pyrin domain containing 3
NLRs: Nucleotide-binding and oligomerization domain (NOD)-like receptors
OS: Overall survival
OSCC: Oral squamous cell carcinoma
PRRs: Pattern recognition receptors
rIL-1β: Recombinate interleukin-1β
ROS: Reactive oxygen species
shRNA: Short hairpin RNA
TLR: Toll-like receptor
